# Cloning of the *Arabidopsis SMAP2* promoter and analysis of its expression activity

**DOI:** 10.1038/s41598-024-61525-1

**Published:** 2024-05-20

**Authors:** Anar Bao, Tongtong Jiao, Ting Hu, Kai Cui, Weijie Yue, Yanxi Liu, Hua Zeng, Jinhong Zhang, Shining Han, Ming Wu

**Affiliations:** 1 College of Life Sciences, Jilin Agricultural University, Changchun, 130118 People’s Republic of China; 2 TECON Pharmaceutical Co., Ltd., Suzhou, 215000 People’s Republic of China

**Keywords:** Biochemistry, Biological techniques, Molecular biology, Plant sciences

## Abstract

The *SMALL ACIDIC PROTEIN (SMAP)* gene is evolutionarily indispensable for organisms. There are two copies of the *SMAP* gene in the *Arabidopsis thaliana* genome, namely, *SMAP1* and *SMAP2*. The function of SMAP2 is similar to that of SMAP1, and both can mediate 2,4-D responses in the root of *Arabidopsis*. This study cloned the *AtSMAP2* genetic promoter sequence. Two promoter fragments of different lengths were designed according to the distribution of their *cis*-acting elements, and the corresponding β- glucuronidase (GUS) expression vector was constructed. The expression activity of promoters of two lengths, 1993 bp and 997 bp, was studied by the genetic transformation in *Arabidopsis*. The prediction results of *cis*-acting elements in the promoter show that there are many hormone response elements in 997 bp, such as three abscisic acid response elements ABRE, gibberellin response elements P-box and GARE-motif and auxin response element AuxRR-core. Through GUS histochemical staining and qRT‒PCR analysis, it was found that the higher promoter activity of P_AtSMAP2-997_, compared to P_AtSMAP2-1993_, drove the expression of *GUS* genes at higher levels in *Arabidopsis*, especially in the root system. The results provide an important basis for subsequent studies on the regulation of *AtSMAP2* gene expression and biological functions.

## Introduction

During the growth and development of plants, genes are expressed in different growth and development stages and in different tissues and the promoter of the gene itself plays a key role in this process. A promoter is a segment of DNA sequence located upstream of the gene that provides recognition and binding by RNA polymerase. Transgenic technology is often used in the functional study of specific genes or in molecular breeding techniques that enable recipient organisms to acquire specific traits. The accurate expression of the promoter of the target gene is particularly important in this process, as sometimes even the same target gene is expressed differently depending on its genetic characteristics. According to expression characteristics, promoters can be categorized as constitutive promoters, inducible promoters, and tissue-specific promoters. The selection of an appropriate promoter is important for the foreign gene to perform the desired function. In most transgenic engineering, constitutive promoters are often used to drive the expression of exogenous genes due to their independence from temporal and spatial constraints and the induction of substances, as well as their advantages of being efficient and more stable, such as the cauliflower virus (CaMV) *35S* promoter^1^ and ubiquitin 1 (Ubi) promoter^2,3^. Inducible promoters do not transcribe or have low transcriptional activity under normal circumstances but can effectively regulate the expression of downstream genes when induced by specific biological factors or physical and chemical substances. At the same time, this type of promoter can reduce the damage caused by the expression of foreign genes in transgenic plants, such as the *rbcs* promoter^4^. Tissue-specific promoters have the characteristics of driving the selective expression of target genes in specific cells, tissues, or developmental stages and are commonly used to regulate plant growth and development^5^, such as cotton fiber-specific *GhPRP5*^6^ and tomato fruit-specific *E8* promoter^7^. Several studies have demonstrated that promoters play a key role in gene expression and regulation in plants. Promoters mainly regulate the initiation time, tissue specificity, and expression intensity of gene expression^8,9^. It can also combine with corresponding transcription factors and other co-factors to precisely control the efficiency of gene expression, respond to various biotic and abiotic stresses, and initiate various life activities^10,11^. Therefore, the study of gene promoters is of great significance for understanding the expression characteristics of genes.

The phytohormone auxin regulates plant development by inducing cellular responses and altering gene expression. 2,4-dichlorophenoxyacetic acid (2,4-D) is a functional analog of the plant endogenous auxin indole-3-acetic acid (IAA), which is often used as a source of auxin and can induce auxin-related responses, such as suppression of root growth and induction of lateral root growth^12^. IAA is unstable under blue light and UV light^13^, and 2,4-D has greater stability in growth media, so in various physiological experiments, compared with IAA, 2,4-D is more suitable as an exogenous source of auxin. The difference between IAA and 2,4-D is in mode of transport and metabolism; 2,4-D is excreted and broken down more slowly than IAA and therefore accumulates more in the cells^14–17^.

Studies have shown that auxin signaling is mainly accomplished through the degradation of the AUXIN/IAA (AUX/IAA) repressor proteins, which is dependent on the ubiquitin‒proteasome degradation pathway^18^. The degradation of AUX/IAA is directly regulated by SCF^TIR1/AFB^ complex, consisting of CULLIN 1 (CUL1), S PHASE KINASE-ASSOCIATED PROTEIN 1 (SKP1), RING-BOX PROTEIN 1 (RBX1) and a substrate-recognizing F-box protein, TRANSPORT INHIBITOR RESPONSE 1 (TIR1) or AUXIN SIGNALING F-BOXES (AFBs)^19^. Auxin binds to TIR1/AFB to promote SCF^TIR1/AFB^ with AUX/IAA proteins and the ubiquitinated AUX/IAA protein is degraded by the 26S proteasome. The AUX/IAA protein and auxin response factors (ARFs) form heterodimers and inhibit the transcriptional activity of ARFs^20^. ARFs can specifically bind to the auxin response element (AuxRE) "TGTCTC" in the promoter region of auxin-responsive genes to activate or inhibit gene expression^21^. Therefore, the degradation of AUX/IAA proteins leads to ARFs release, thereby regulating the expression of downstream genes and activating a series of auxin responses.

To better understand the mechanisms of auxin signaling, mutants with altered auxin responses have been screened using a variety of genetic approaches; however, due to the absolute necessity of auxin for embryogenesis, seedling mutants treated with auxin screening have certain limitations. Another approach is to screen *Arabidopsis* mutants for abnormal responses to compounds that alter or antagonize the auxin response. Therefore, studies have reported that using an inhibitor of auxin signaling events, *p*-chlorophenoxyisobutyric acid (PCIB), to screen *Arabidopsis* mutants, and finally obtained an *anti-auxin resistant* (*aar*) mutant, this mutant can still grow longer roots on PCIB-containing medium^22,23^. In one of the mutants, *aar1-1*, in terms of root elongation, lateral root formation, seed germination in the presence of abscisic acid, and degradation of AUX/IAA proteins, the response to endogenous auxin IAA is the same as that of the wild type but shows specific resistance to the synthetic auxin 2,4-D, while having no effect on 2,4-D transport or metabolism^24^. Molecular characterization of this mutant showed that the *SMALL ACIDIC PROTEIN1* (*SMAP1*) gene confers 2,4-D and PCIB resistance and a longer hypocotyl phenotype^23,24^. The SMAP1 protein consists of 62 amino acids with a size of 6.9 kDa and an isoelectric point of 3.4. Although the SMAP protein has no known functional domains, it has a highly conserved 18 amino acid sequence rich in phenylalanine and aspartic acid at its C-terminal region^24^. Studies of *aar1* mutants and the *SMAP1* gene show that the SMAP1 protein functions upstream of AUX/IAA protein degradation in auxin signaling and physically interacts with the constitutive photomorphogenic9 signalosome (CSN) through its F/D region^24,25^. CSN is a key regulator of cullin-RING ubiquitin ligases, the largest family of E3 ubiquitin ligases, including SCF^TIR1/AFB^, the major upstream pathway that regulates auxin signaling^26^. This F/D region is present in the genomes of various plants and animals, which means that *SMAP* genes are evolutionarily indispensable to organisms^24,27^.

There is another copy of the *SMAP* gene in the *Arabidopsis* genome, *SMALL ACIDIC PROTEIN 2 (SMAP2)*. The SMAP2 protein consists of 72 amino acids with a size of 9.4 kDa and an isoelectric point of 3.5. SMAP1 and SMAP2 share 43.5% identity in the entire amino acid sequence and 83.3% identity in their highly conserved C-terminal F/D-rich domains^27^. While there are two *SMAP* genes in the *Arabidopsis* genome, a BLAST search revealed only one *SMAP* gene in other dicots, such as populus, grape, and tomato^27^. RNA expression analysis showed that *SMAP1* mRNA was expressed in the whole *Arabidopsis* plant, while *SMAP2* mRNA was only expressed in siliques and anthers, with a high level of expression in anthers and a small amount in green siliques^27^. By overexpressing the *SMAP1* and *SMAP2* genes, respectively, in *aar1* mutants, the function of SMAP2 is similar to that of SMAP1, and both can mediate the 2,4-D response in *Arabidopsis* roots^27^.

However, there are few studies on the other functions of the *SMAP2* gene and its expression regulation mechanism, and studies on its promoter activity have not been reported. In this study, the promoter sequence of the *AtSMAP2* gene was cloned, and two promoter fragments of different lengths were designed according to the distribution of its *cis*-acting elements, and the corresponding β-glucuronidase (*GUS*) expression vector was constructed. The expression activities of the two lengths of promoter, which were 1993 bp and 997 bp respectively, were investigated by genetic transformation in *Arabidopsis*. Through GUS histochemical staining and qRT‒PCR analysis, it was found that the higher promoter activity of P_AtSMAP2-997_, compared to P_AtSMAP2-1993_, drove the expression of *GUS* genes at higher levels in *Arabidopsis*, especially in the root system.

## Results

### Sequence analysis of the *AtSMAP2* promoter

**Figure 1:**
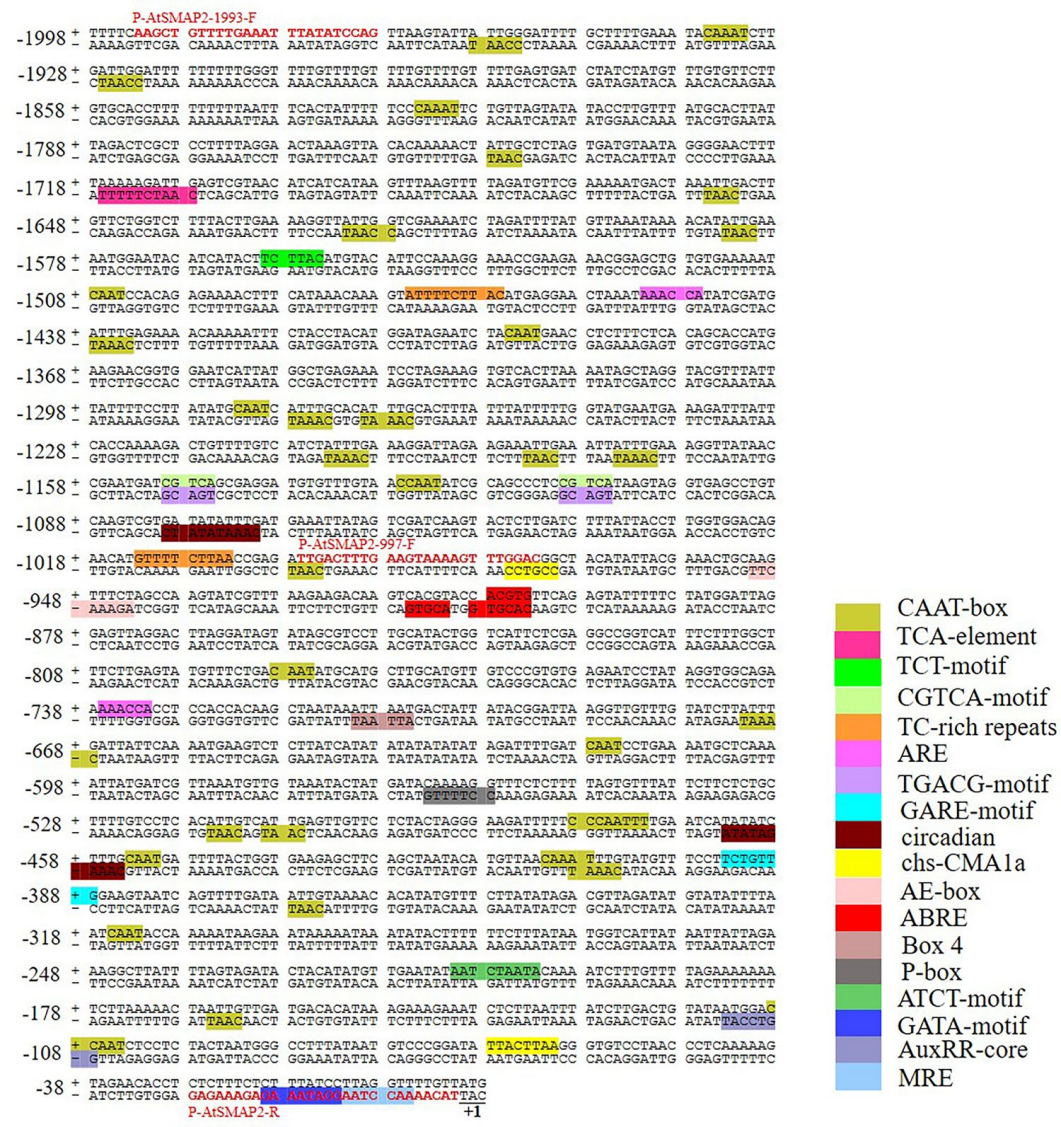
Sequence analysis of full-length promoter of *AtSMAP2* gene. The translation initiation site ‘ATG’ (marked in underline) of *AtSMAP2* was numbered as + 1. The *cis*-acting elements of the 1998 bp full-length promoter sequence upstream of ATG were predicted by searching the PLACE and PlantCARE databases. The different *cis*-acting elements were marked in different colours. Among these *cis*-acting elements, CAAT-box is the major *cis*-regulatory elements; TCA-element, CGTCA-motif, TGACG-motif, ABRE, P-box, GARE-motif, AuxRR-core belong to the phytohormone response elements; TC-rich repeats, ARE, circadian belong to the environmental stress regulation-related elements; TCT-motif, chs-CMA1a, AE-box, Box 4, ATCT-motif, GATA-motif, MRE belong to the light response elements. The bold sequences in red are the forward primer sequences used to clone the 1993 bp and 997 bp promoter fragments and the common reverse primer sequences, respectively.

The 1998 bp promoter sequence upstream of the transcriptional start codon ATG was obtained from the *AtSMAP2* gene (AT3G24280) sequence on The *Arabidopsis* Information Resource (TAIR) (https://www.arabidopsis.org/). The *cis*-acting elements of the 1998 bp promoter sequence upstream of ATG (the translation initiation site ATG is numbered as + 1) were predicted by searching the PLACE and PlantCARE databases (Fig. 1). Using these two types of analysis software, four types of *cis*-acting elements were found to be present in this sequence, namely major *cis*-regulatory elements, phytohormone response elements, light response elements, and environmental stress regulation-related elements. The core sequences and predicted functions of the various *cis*-acting elements are listed in Table 1. The prediction results showed that multiple hormone signal response elements were concentrated in the 997 bp fragment, including three abscisic acid response elements, two gibberellin response elements, and one auxin response element. It is speculated that *AtSMAP2* gene expression may be related to the hormone response.

**Table 1:** Bioinformatics analysis of the *AtSMAP2* promoter sequence.

Component name	Core sequence	Positions	Predictive function	Kind
TATA-box	TATA	− 83, − 203, 326, − 463, − 565, − 630, − 750, − 858, − 1062, − 1288, − 1809, − 1976 etc., a total of 56 positions	Core promoter element around -30 of transcription start	Major *cis*-regulatory elements
CAAT-box	CAAT	− 109, − 409, − 412, − 479, − 510, − 516, − 672, − 1074, − 1126, − 1174, − 1203, − 1269, − 1277, − 1438, − 1621, − 1824, − 1928, − 1935, − 1959	Common cis-acting element in promoter and enhancer regions	Major *cis*-regulatory elements
ABRE	ACGTG	− 916, − 908	Cis-acting element involved in the abscisic acid responsiveness	Phytohormone response elements
ABRE	CACGTG	− 909	Cis-acting element involved in the abscisic acid responsiveness	Phytohormone response elements
P-box	CCTTTTG	− 564	Gibberellin-responsive element	Phytohormone response elements
GARE-motif	TCTGTTG	− 394	Gibberellin-responsive element	Phytohormone response elements
AuxRR-core	GGTCCAT	− 114	Cis-acting regulatory element involved in auxin responsiveness	Phytohormone response elements
TCA-element	CCATCTTTTT	− 1717	Cis-acting element involved in salicylic acid responsiveness	Phytohormone response elements
TGACG-motif	TGACG	− 1150, − 1110	Cis-acting regulatory element involved in the meja-responsiveness	Phytohormone response elements
TC-rich repeats	GTTTTCTTAC	− 1013, − 1476	Cis-acting element involved in defense and stress responsiveness	Environmental stress regulation-related elements
ARE	AAACCA	− 1452, − 737	Cis-acting regulatory element essential for the anaerobic induction	Environmental stress regulation-related elements
circadian	CAAAGATATC	− 1080, − 464	Cis-acting regulatory element involved in circadian control	Environmental stress regulation-related elements
MRE	AACCTAA	− 12	MYB binding site involved in light responsiveness	Light response elements
GATA-motif	AAGGATAAGG	− 20	Part of a light responsive element	Light response elements
chs-CMA1a	TTACTTAA	− 68	Part of a light responsive element	Light response elements
ATCT-motif	AATCTAATCC	− 211	Part of a conserved DNA module involved in light responsiveness	Light response elements
Box 4	ATTAAT	− 711	Part of a conserved DNA module involved in light responsiveness	Light response elements
AE-box	AGAAACTT	− 951	Part of a module for light response	Light response elements
TCT-motif	TCTTAC	− 1560	Part of a light responsive element	Light response elements

### Generation of *GUS*-expressing transgenic *Arabidopsis *driven by different *AtSMAP2* promoter fragments

**Figure 2:**
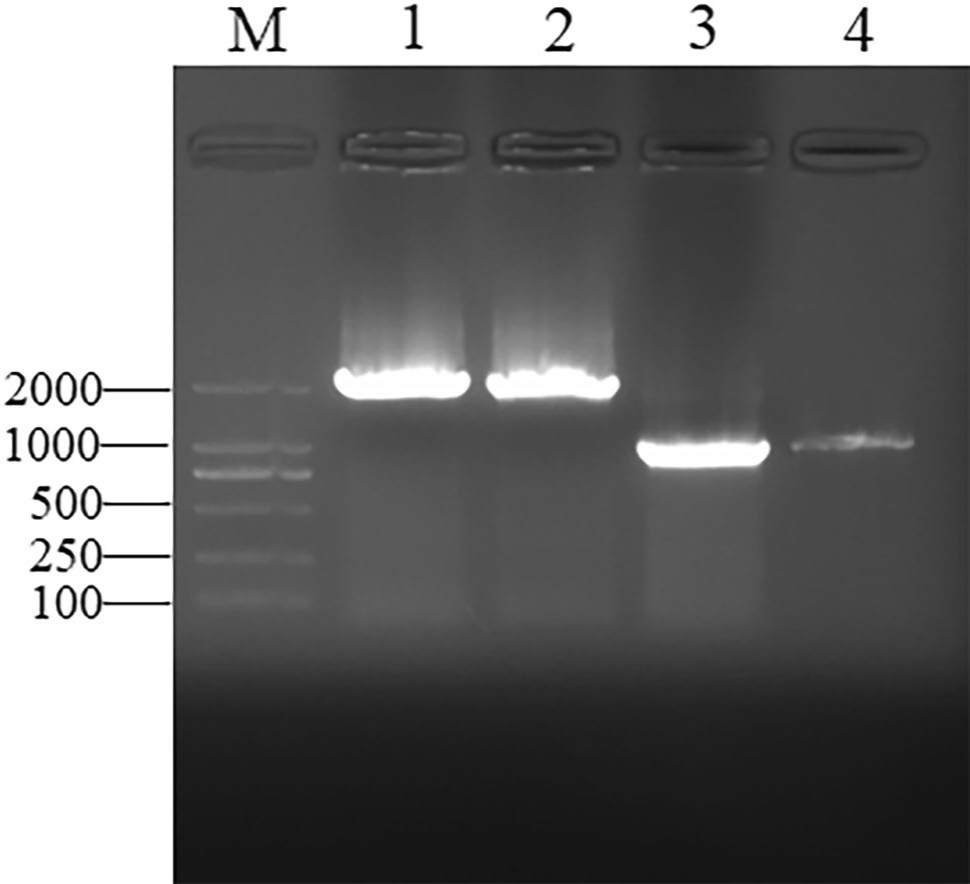
Cloning of promoter region of *AtSMAP2*. M: DNA marker (100–2000 bp); lane 1,2: P_AtSMAP2-1993_; lane 3,4: P_AtSMAP2-997_.

Based on the results obtained from *cis*-acting element prediction of the promoter sequence upstream of ATG of the *AtSMAP2* gene using PLACE and PlantCARE, a 1993 bp promoter fragment containing most of the *cis*-acting elements upstream of this gene and a 997 bp promoter fragment upstream of this gene containing multiple hormone response elements, namely 3 abscisic acid response elements, 2 gibberellin response elements, and 1 auxin response element, were amplified by two specific forward PCR primers and one common reverse primer (Fig. 2). To determine the expression activity of the 1993 bp and 997 bp promoters, the PCR product was fused with the *GUS* reporter gene and used for *Agrobacterium*-mediated *Arabidopsis* genetic transformation.

**Figure 3:**
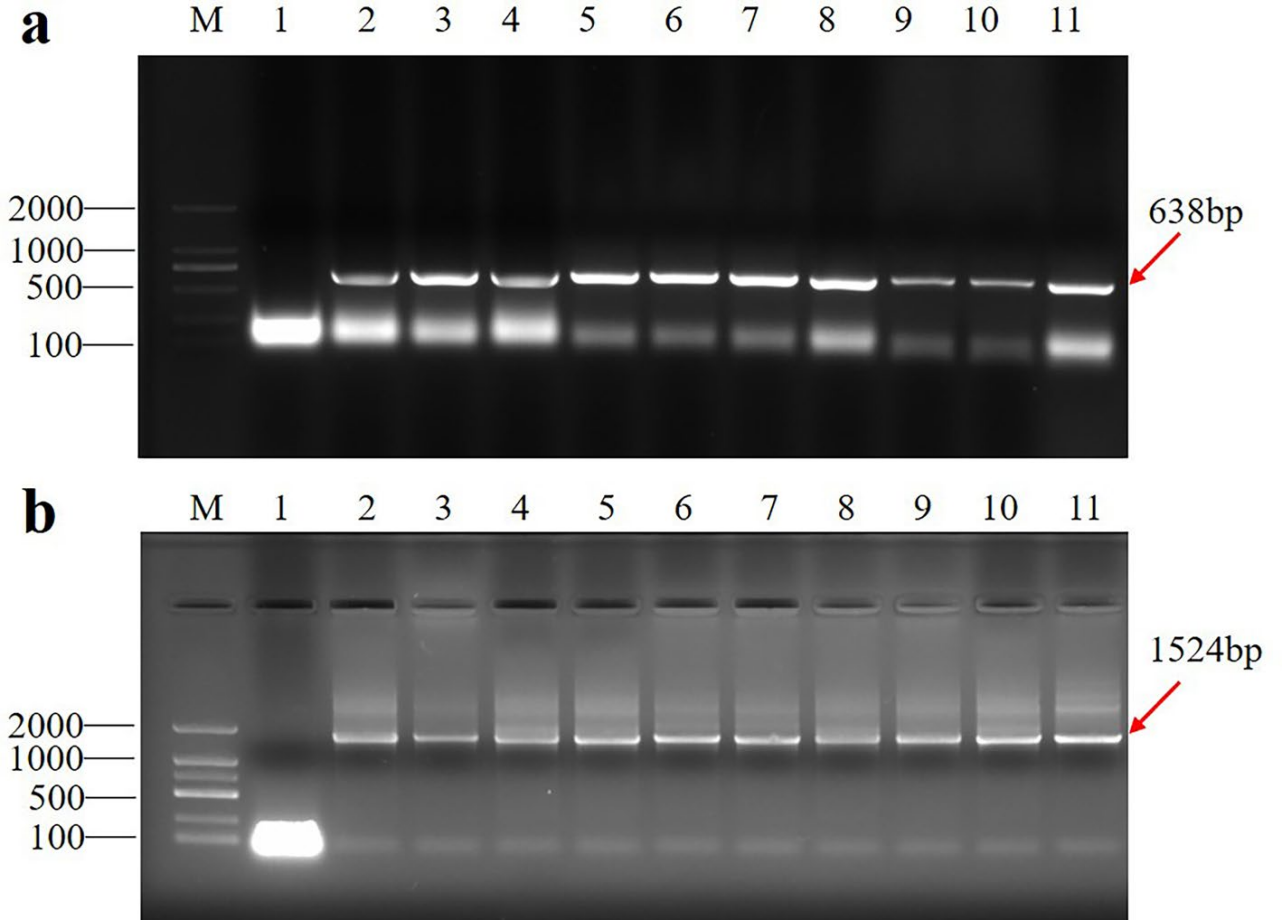
PCR analysis of transgenic *A. thaliana* events showing two *promoter-GUS* fragments. (**a**) PCR identification of P_AtSMAP2-997_-GUS strain. (**b**) PCR identification of P_AtSMAP2-1993_-GUS strain. M: DNA marker (100-2000 bp); lane 1: untransformed negative control, lane 2: pMDC162 plasmid DNA, lane 3 to11: transgenic plants of *A. thaliana.*

T1 generation transgenic *Arabidopsis* seeds were screened on an MS medium containing 50 mg/L hygromycin. After extracting DNA from a small number of leaves of the T1 generation resistant plants, PCR detection was performed with *promoter-GUS*-specific primers, and the bands were amplified to the same size as the positive control target band, indicating that the two *AtSMAP2* promoter fragments were successfully integrated into the Col-0 genome (Fig. 3). At least 20 individual lines with a single transgene insertion were selected in the T2 generation based on the 3:1 segregation ratio for hygromycin resistance. Through the evaluation of the segregation ratio, the T3 transgenes homozygotes were confirmed from a single generation of T2 plants and used for further histochemical staining and qRT‒PCR analysis. A number of independent transgenic lines were obtained, and at least 5 typical GUS-positive independent T3 lines were analyzed by histochemical staining. Histochemical staining of transgenic *Arabidopsis* for GUS revealed that the two promoter fragments always had different activities, while the GUS expression was consistent under the same promoter fragment.

### Expression patterns of two *AtSMAP2* promoter fragments in different growth stages and tissues

**Figure 4:**
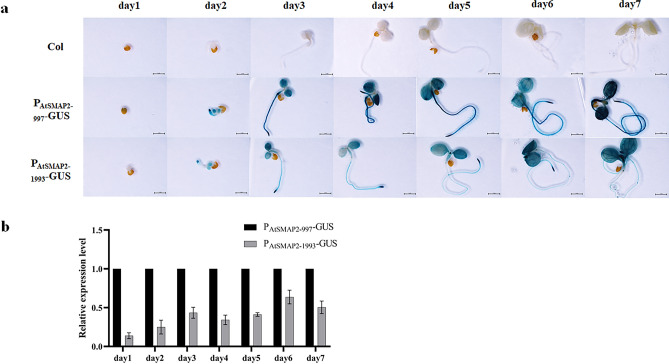
Detection of GUS expression driven by different *AtSMAP2* promoter fragments in transgenic *Arabidopsis* plants at the seedling stage. (**a**) GUS histochemical assay in transgenic *Arabidopsis* seedlings. Bars = 1 mm. (**b**) qRT-PCR detection of *GUS* expression patterns in transgenic *Arabidopsis* plants at the seedling stage. Data were presented as the mean ± SE of three separate measurements for each independent line.

To understand the expression activities of the two promoters, GUS staining was performed at the seedling stage of the transgenic positive lines, and staining was performed every 24 h (Fig. 4a). The 24 h staining results showed that for the P_AtSMAP2-997_-GUS strain, the dyeing effect was more obvious, and for the P_AtSMAP2-1993_-GUS strain, the dyeing effect was not obvious. The staining results of seedlings on 2 days of germination showed that the roots and leaves of the P_AtSMAP2-997_-GUS line were stained, and the leaves and root tips of the P_AtSMAP2-1993_-GUS strain were also stained, but their expression activity was lower. The staining results of seedlings on the 3rd and 4th day of germination showed that both the P_AtSMAP2-997_-GUS and P_AtSMAP2-1993_-GUS strains were stained throughout, but the P_AtSMAP2-1993_-GUS strain had lower expression activity in leaves, hypocotyls, and roots than the P_AtSMAP2-997_-GUS strain. The staining results from the 5th day to the 7th day of germination showed that unlike the P_AtSMAP2-1993_-GUS line, the entire P_AtSMAP2-997_-GUS strain was stained and its root hairs were also stained, in addition to significantly stronger staining of its primary roots.

**Figure 5:**
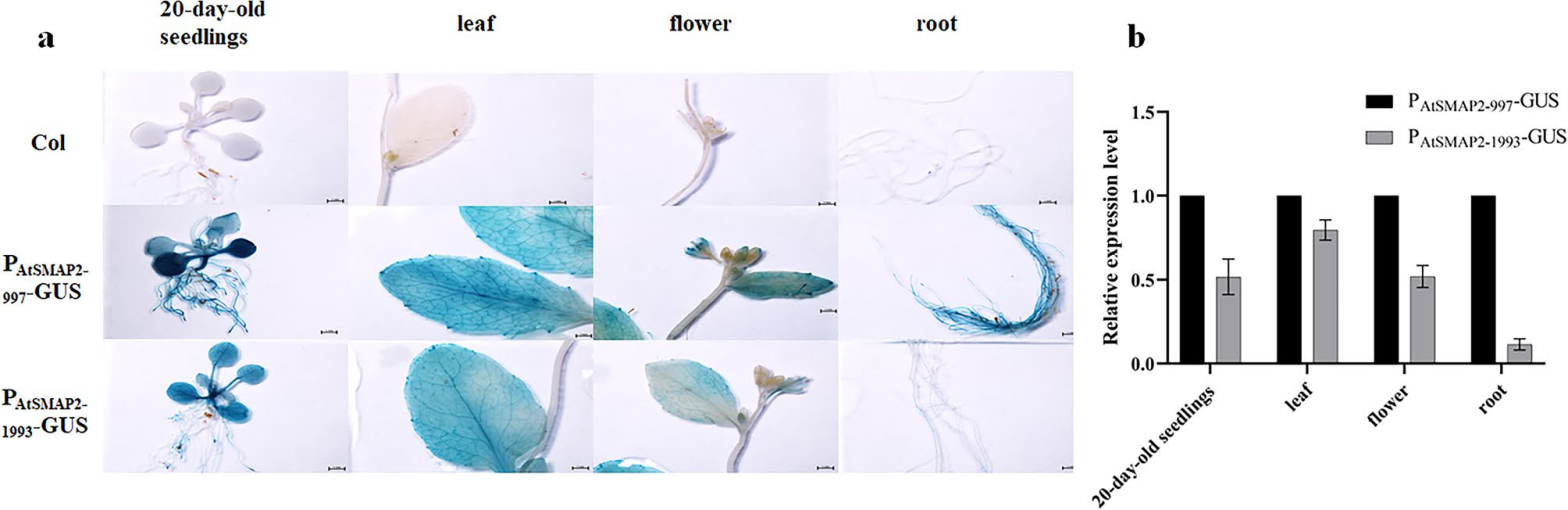
Expression patterns of *AtSMAP2* promoter fragments in different developmental stages and tissues. (**a**) GUS histochemical assay in 20-day-old seedlings and different mature organs of transgenic *Arabidopsis*. Bars = 1 mm. (**b**) qRT-PCR detection of *GUS* expression patterns in 20-day-old seedlings and different mature organs of transgenic *Arabidopsis*. Data were presented as the mean ± SE of three separate measurements for each independent line.

GUS staining was also performed in 20-day-old transgenic *Arabidopsis* lines and mature organs of transgenic *Arabidopsis*. The leaves, flowers, and roots of mature transgenic *Arabidopsis* lines were stained and observed. Histochemical staining analysis showed that (Fig. 5a), consistent with the staining results at the seedling stage, root staining was stronger in both the 20-day-old and mature stage of the P_AtSMAP2-997_-GUS strain than in the P_AtSMAP2-1993_-GUS strain. The staining results at the 20-day-old plants showed that in the P_AtSMAP2-997_-GUS strain, the degree of staining of leaves was not uniform, and the first pair of leaf primordia and hypocotyl showed a stronger degree of staining than the P_AtSMAP2-1993_-GUS strain, while the second pair of leaf primordia was lighter stained than those of the P_AtSMAP2-1993_-GUS strain. There was no significant difference in the degree of staining of leaves at the mature stage of the two lines, and the degree of staining of flowers of the P_AtSMAP2-997_-GUS line was stronger than that of the P_AtSMAP2-1993_-GUS line.

To validate the results of GUS staining, a highly sensitive qRT‒PCR analysis of *GUS* gene transcription level was further used to verify the activity of the two *AtSMAP2* promoters with different fragment lengths. The qRT‒PCR results were consistent with the GUS histochemical staining results. From the first day of germination to the seventh day of germination, the promoter activity of P_AtSMAP2-997_ was always stronger than that of P_AtSMAP2-1993_ (Fig. 4b). Comparing different tissues, it was found that the difference between the two promoters was most pronounced in the roots at the 20-day-old and mature stage of *Arabidopsis thaliana*, with P_AtSMAP2-997_ promoter activity consistently stronger than P_AtSMAP2-1993_ activity in roots (Fig. 5b).

To sum up the results, the higher promoter activity of P_AtSMAP2-997_, compared with P_AtSMAP2-1993_, drives the expression of the *GUS* gene at a higher level in *Arabidopsis thaliana*, especially in the root system where the expression difference is more obvious, suggesting that there are DNA sequence elements in the range of -1 bp ~ -997 bp to ensure the expression of *AtSMAP2* gene at a higher level, while there may be sequences that inhibit its expression in the range of − 997 ~ − 1993 bp.

### Expression activity of the two *AtSMAP2* promoter fragments under different concentrations of 2,4-D and IAA

**Figure 6:**
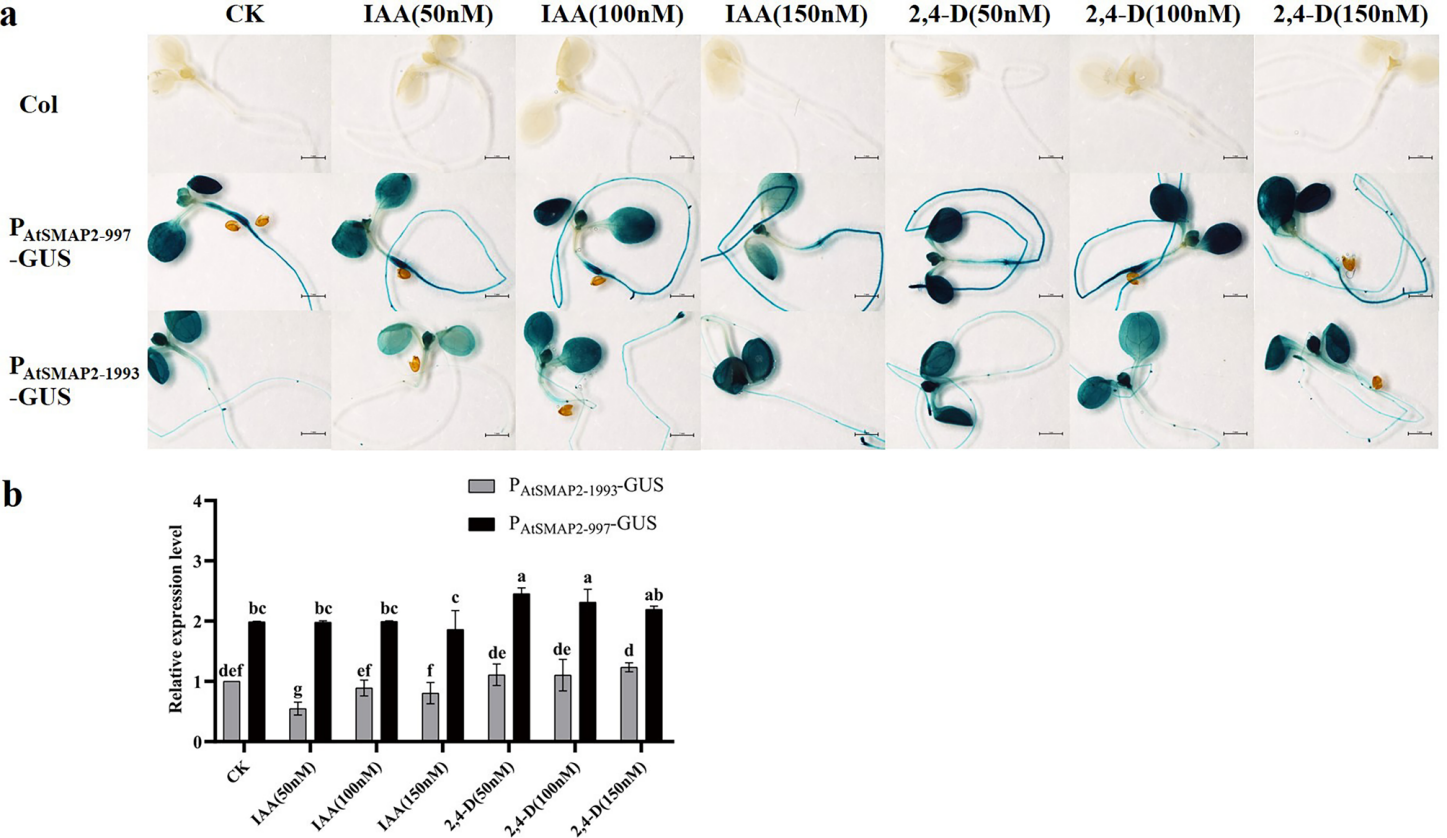
Detection of GUS expression driven by different *AtSMAP2* promoter fragments in transgenic *Arabidopsis* treated with different concentrations of the exogenous hormone 2,4-D and IAA. (**a**) GUS histochemical analysis of transgenic *Arabidopsis* after treatment with 2,4-D and IAA. Bars = 1 mm. (**b)** qRT-PCR detection of GUS expression in transgenic *Arabidopsis* seedlings treated with 2,4-D and IAA. Data were presented as the mean ± SE of three separate measurements for each independent line. Different lower-case letters above the bars indicated significantly different at* p* < 0.05.

Both P_AtSMAP2-997_-GUS and P_AtSMAP2-1993_-GUS lines were stained after treatment with exogenous hormone 2,4-D and IAA. After treatment with the exogenous hormone IAA, the depth of GUS staining in roots and leaves of the P_AtSMAP2-997_-GUS strains did not change significantly as a result of the increase in its concentration (Fig. 6a). The P_AtSMAP2-1993_-GUS strain showed a significant decrease in the depth of staining in roots and leaves at 50 nM IAA treatment, whereas no significant change in the depth of staining occurred under 100 nM and 150 nM IAA treatment conditions (Fig. 6a). Notably, the P_AtSMAP2-997_-GUS strain always stained darker than the P_AtSMAP2-1993_-GUS strain under the three concentrations of IAA treatment (Fig. 6a). The P_AtSMAP2-997_-GUS strain showed enhanced expression activity in the roots and leaves at all three concentrations of 2,4-D treatment compared to the control group (Fig. 6a). The P_AtSMAP2-1993_-GUS strain was also stained under all three concentrations of 2,4-D treatment, but its expression activity in roots and leaves did not differ from that of its control strain without the hormone (Fig. 6a). After 2,4-D treatment, the expression activity of the P_AtSMAP2-1993_-GUS strain remained significantly lower than that of the P_AtSMAP2-997_-GUS strain (Fig. 6a).

The qRT-PCR results were consistent with the GUS histochemical staining results that the activity of the P_AtSMAP2-997_ promoter was always significantly higher than that of the P_AtSMAP2-1993_ promoter, regardless of the addition of IAA or 2,4-D (Fig. 6b). The expression activity of the P_AtSMAP2-997_ promoter was not significantly different from that of the control after the addition of the exogenous hormone IAA, whereas the expression activity of the P_AtSMAP2-1993_ promoter was significantly lower than that of the control under the 50 nM IAA treatment condition (Fig. 6b). The expression activity of P_AtSMAP2-997_ initiated by the addition of both 50 nM and 100 nM exogenous hormone 2,4-D was significantly higher than that of the control, whereas the P_AtSMAP2-1993_ promoter was not significantly different from that of the control at any of the three 2,4-D concentrations (Fig. 6b). In summary, in *Arabidopsis*, the *SMAP2* gene promoter showed different responses to IAA and 2,4-D, also, the activity of the P_AtSMAP2-997_ promoter was consistently stronger than that of the P_AtSMAP2-1993_ promoter in this process.

### Expression activity of the two *AtSMAP2* promoter fragments under different concentrations of ABA

**Figure 7:**
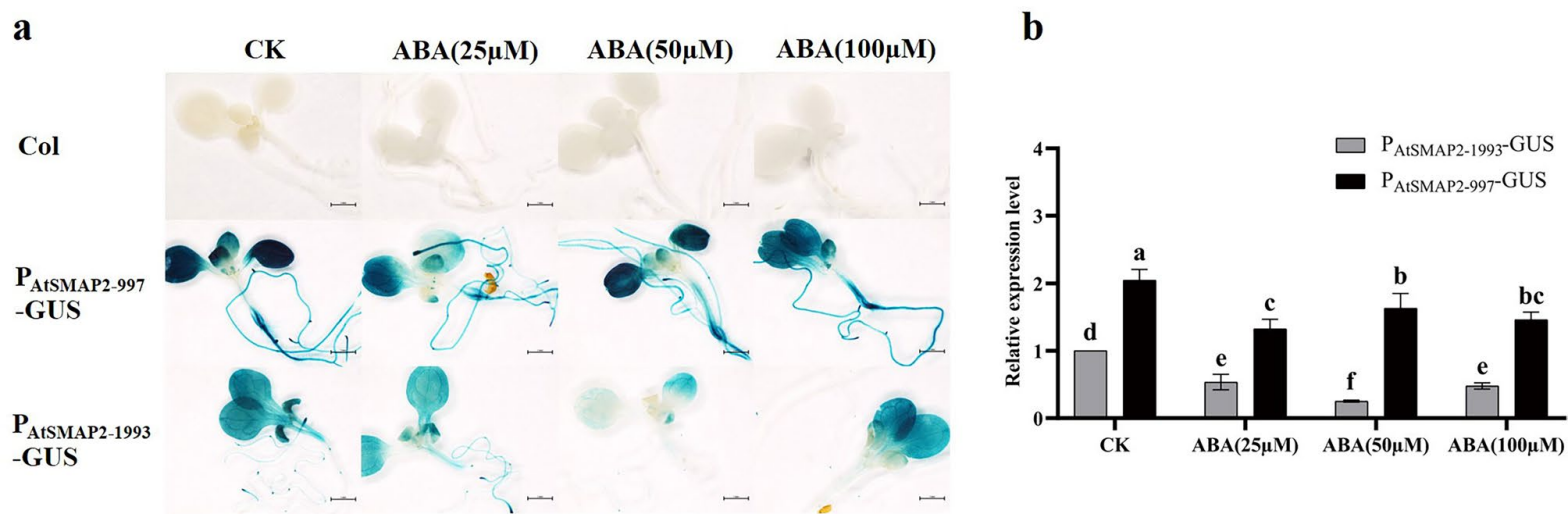
Detection of GUS expression driven by different *AtSMAP2* promoter fragments in transgenic *Arabidopsis* treated with different concentrations of the exogenous hormone ABA. (**a**) GUS histochemical analysis of transgenic *Arabidopsis* after treatment with ABA. Bars = 1 mm. (**b**) qRT-PCR detection of GUS expression in transgenic *Arabidopsis* seedlings treated with ABA. Data were presented as the mean ± SE of three separate measurements for each independent line. Different lower-case letters above the bars indicated significantly different at* p* < 0.05.

The addition of three concentrations of exogenous ABA, 25 µM, 50 µM and 100 µM, respectively, resulted in reduced GUS expression activity in the leaves of the P_AtSMAP2-997_-GUS strain compared to its control, as well as in its roots at 25 µM and 50 µM (Fig. 7a). Observation of GUS expression activity in the P_AtSMAP2-1993_-GUS strain revealed that it exhibited reduced GUS expression activity in both leaves and roots compared to its control at 50 µM and 100 µM ABA (Fig. 7a). The qRT-PCR results showed that the three concentrations of ABA treatment exhibited a significant attenuation of GUS expression activity in both P_AtSMAP2-997_-GUS and P_AtSMAP2-1993_-GUS lines compared to their controls (Fig. 7b). Therefore, it was hypothesized that the expression activity of the *SMAP2* gene promoter would be regulated by ABA, which inhibits its promoter expression activity. Finally, it is worth noting that the activity of the P_AtSMAP2-997_ promoter remained consistently stronger than that of the P_AtSMAP2-1993_ promoter when exogenous ABA was added (Fig. 7b).

### Expression activity of the two *AtSMAP2* promoter fragments under different concentrations of MeJA

**Figure 8:**
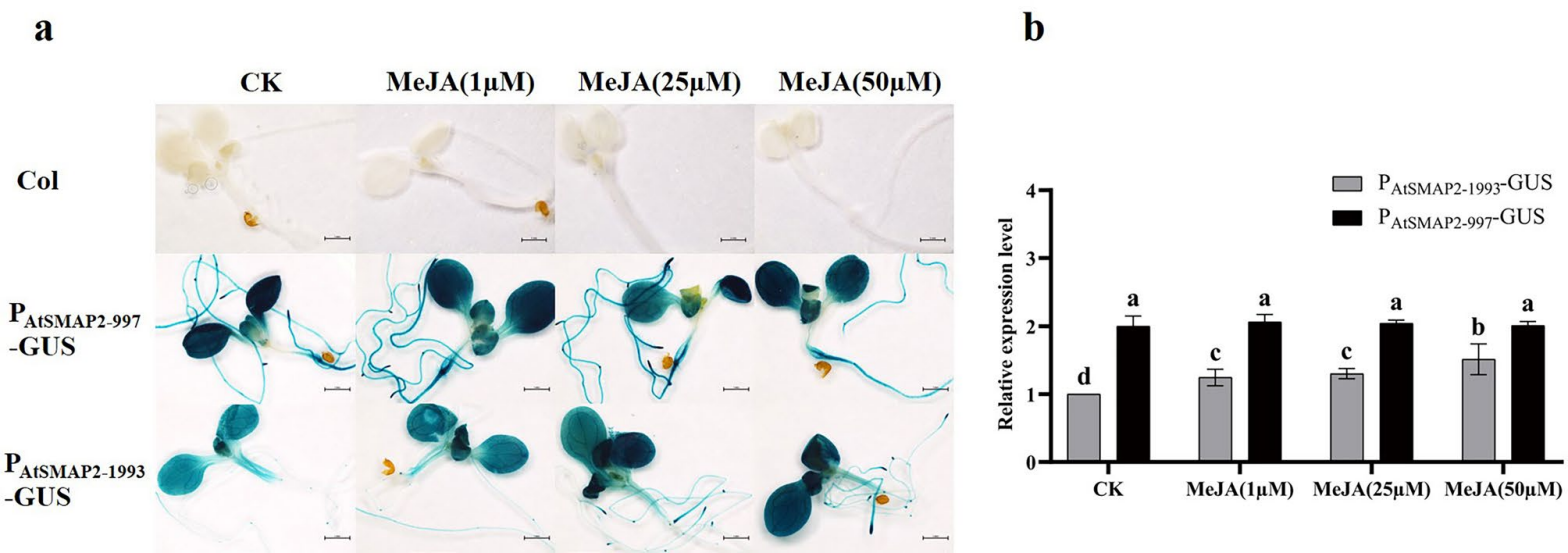
Detection of GUS expression driven by different *AtSMAP2* promoter fragments in transgenic *Arabidopsis* treated with different concentrations of MeJA. (**a**) GUS histochemical analysis of transgenic *Arabidopsis* after treatment with MeJA. Bars = 1 mm. (**b**) qRT-PCR detection of GUS expression in transgenic *Arabidopsis* seedlings treated with MeJA. Data were presented as the mean ± SE of three separate measurements for each independent line. Different lower-case letters above the bars indicated significantly different at* p* < 0.05.

After treating *Arabidopsis* seedlings with three concentrations of exogenous MeJA, 1 µM, 25 µM, and 50 µM, respectively, seedlings of the P_AtSMAP2-997_-GUS strain were observed, and none of them showed changes in GUS expression activity in the whole plant compared to its control (Fig. 8a). Seedlings of the P_AtSMAP2-1993_-GUS strain were observed and found to exhibit stronger GUS expression activity than the corresponding controls when all three concentrations of MeJA were added (Fig. 8a). The qRT-PCR results showed that the GUS expression in the P_AtSMAP2-997_-GUS strain, after the addition of the three concentrations of MeJA, was not significantly different from that of its control, whereas the GUS expression in the P_AtSMAP2-1993_-GUS strain showed significant up-regulation in the three different concentrations of the treatment compared to its control (Fig. 8b). Overall, the P_AtSMAP2-1993_ promoter showed sensitivity to exogenous MeJA, but the expression activity of the P_AtSMAP2-997_ promoter was still stronger than that of the P_AtSMAP2-1993_ promoter.

## Discussion

### The 997 bp promoter fragment upstream of the *AtSMAP2* gene is sufficient for efficient expression of this gene

When an exogenous gene is introduced into a recipient plant, the expression must be driven by a promoter. Therefore, in the field of plant engineering, the study of promoters is of great significance to the expression of exogenous genes. In this study, it was found that the *AtSMAP2* gene promoter contains core elements that regulate gene expression, such as the TATA box, which is necessary for RNA polymerase II transcription initiation, and the CAAT box, which enhances gene transcription. In addition, there are hormone response elements, light regulatory elements, stress response elements, etc., indicating that *AtSMAP2* gene expression may be affected by various factors, such as light, hormones, and environmental stress.

In this study, two promoter fragments of different lengths of *AtSMAP2* gene, 997 bp (− 1 to − 997 bp) and 1993 bp (− 1 to − 1993 bp), were designed, and the corresponding GUS expression vector was constructed. The expression activity of two lengths of the promoter was studied by *Arabidopsis* genetic transformation. Notably, this study showed that the GUS expression activity in the P_AtSMAP2-997_-GUS strain was always higher than that in the P_AtSMAP2-1993_-GUS strain. In fact, the 1993 bp promoter fragment contains all the *cis*-acting elements contained in the 997 bp fragment and contains more typical core elements, TATA-box and CATA-box, so it should have higher expression activity than the 997 bp promoter fragment. However, the results of our study were the opposite, in transgenic *Arabidopsis*, the 997 bp promoter fragment always had higher expression activity than the 1993 bp promoter from the germination to maturation stages and under exogenous hormone-addition conditions. It was therefore hypothesized that there are sequences in − 1 to − 997 bp that ensure efficient expression of the *AtSMAP2* gene, while there are sequences in − 998 to − 1993 bp that repress the expression of this gene. For example, the MeJA response elements are located at − 1150 and − 1110 bp, and there is no MeJA response element in the 997 bp fragment, yet the promoter fragment of 997 bp still has higher expression activity than that of 1993 bp when exogenous MeJA is added. It was further demonstrated that there is a *cis*-acting element in − 998 to − 1993 bp that represses the expression of this gene, but the exact location needs to be verified by truncation experiments, which will be the focus of our subsequent work. In addition, which specific sequences in − 1 to − 997 bp are essential for the expression of this gene also still need to be verified by subsequent experiments.

### The *AtSMAP2* gene promoter showed different responses to IAA and 2,4-D

The addition of exogenous IAA or 2,4-D usually results in the inhibition of plant root growth^12^, so it has long been widely believed that IAA and 2,4-D inhibit root growth through a similar mechanism. Later studies reported that IAA and 2,4-D elicit differential responses in regulating root growth. The root growth rate is regulated by two interrelated processes, namely, cell expansion and cell production^28^. 2,4-D mainly affects cell division, while IAA affects cell elongation^29^. In addition, studies have demonstrated that 2,4-D-induced inhibition of root or plant growth is related to cellular actin status^29,30^. 2,4-D can alter actin structure in long-term and short-term assays, and the effects of 2,4-D on actin filament organization and root growth were found to be mediated by synergistic interactions between SMAP1 and SCF^TIR1^ ubiquitin proteasome components, as studied with the 2,4-D-specific mutant *aar1-1* and the ubiquitin–proteasome mutants *tir1-1* and *axr1-12*^31^.

In this study, *cis*-acting element prediction showed an auxin response element, AuxRR-core, at − 108 to − 114 bp upstream of the *AtSMAP2* gene. To investigate whether the *AtSMAP2* gene promoter responds to auxin and whether it responds differentially to 2,4-D and IAA, the P_AtSMAP2-997_-GUS and P_AtSMAP2-1993_-GUS lines were treated with exogenous 2,4-D and IAA, respectively. The results of this study indicate that the P_AtSMAP2-997_ promoter is insensitive to IAA, while it can be induced to express by 2,4-D. The P_AtSMAP2-1993_ promoter is sensitive to IAA, which results in the inhibition of its expression activity, and insensitive to 2,4-D. The two promoters respond differently to both IAA and 2,4-D, but the exact location of their actions and the mechanisms are necessary to be further verified.

### The *AtSMAP2* gene promoter may also control this gene in response to ABA and MeJA

Judging from the predicted results of *cis*-acting elements and the differences in expression between the two promoters, it appears that *AtSMAP2* may respond to abscisic acid, gibberellin, auxin, salicylic acid and MeJA. By overexpressing the *AtSMAP1* and *AtSMAP2* genes in *aar1* mutants, it was found that AtSMAP2 functions similarly to AtSMAP1 and can respond to 2,4-D responses in *Arabidopsis* roots^27^, but how AtSMAP2 responds to other phytohormones remains to be studied.

It has been reported that several inducible genes contain conserved ABRE motifs (ACGT) in their promoter regions^32^. In this study, two ABRE motifs were present upstream of the *AtSMAP2* gene and the P_AtSMAP2-997_-GUS and P_AtSMAP2-1993_-GUS lines were treated with exogenous ABA, which showed that both the P_AtSMAP2-1993_ and P_AtSMAP2-997_ promoters responded to ABA stress. ABA represses the expression activity of the P_AtSMAP2-1993_ and P_AtSMAP2-997_ promoters. MeJA induces the synthesis of defensive compounds and initiates the expression of genes associated with pathogenesis. Therefore, MeJA can be used to combat pathogens, salt stress, drought stress, low temperature, heavy metal stress and toxicity of other elements. In this study, two sites upstream of the *AtSMAP2* gene promoter at -1150 and -1110 were predicted to have MeJA-responsive *cis*-acting elements. Treatment of P_AtSMAP2-997_-GUS and P_AtSMAP2-1993_-GUS strains by exogenous MeJA showed that the P_AtSMAP2-997_ promoter was insensitive to exogenous MeJA, whereas the P_AtSMAP2-1993_ promoter showed sensitivity to exogenous MeJA, which was caused by the probable reason that the P_AtSMAP2- 997_ promoter sequence does not contain MeJA-responsive *cis*-acting elements. Gene expression patterns may be a direct indicator of the role of promoters in stress and development. Both ABA and MeJA are phytohormones associated with plant stress response and the *AtSMAP2* gene promoter exhibits a corresponding response to both of them, so it is hypothesized that the properties of this promoter may confer a role to the *AtSMAP2* gene related to stress resistance.

## Materials and methods

### Experimental materials

The *Arabidopsis thaliana* ecotype Col-0, *Agrobacterium* strain GV3101, *Escherichia coli* strains TOP10 and DB3.1, and the plant expression vector pMDC162 plasmid used in this study were all provided by our laboratory. TOPO vectors (pENTR™ Directional TOPO^®^ Cloning Kits) were purchased from Life Technologies, ClonExpress^®^ II One Step Cloning Kit C112 was purchased from Vazyme, and GUS staining kits were purchased from Solarbio.

### *AtSMAP2* gene promoter sequence acquisition and bioinformatics analysis

According to the sequence of the *AtSMAP2* gene (AT3G24280) in The *Arabidopsis* Information Resource (TAIR) (https://www.arabidopsis.org/), the fragment was obtained as a 1998 bp full-length promoter sequence upstream of the transcription initiation codon ATG. This sequence was analyzed using the online tools Plant *Cis*-Acting Regulatory DNA Elements (PLACE) (https://www.dna.afrc.go.jp/PLACE/?action=newplace)^33^ and Plant *Cis*-Acting Regulatory Element (PlantCARE) (http://bioinformatics.psb.ugent.be/webtools/plantcare/html/)^34^ for the identification of *cis*-acting elements in the putative promoter.

### Cloning of the *AtSMAP2* promoter

Genomic DNA was extracted from fresh leaves of wild-type Arabidopsis Col-0 by the CTAB method^35^. Based on the prediction of the *cis*-acting element of the 1998 bp sequence upstream of the *AtSMAP2* gene transcription start codon ATG, the 997 bp and 1993 bp sequences were intercepted as promoter sequences, respectively. The primers were designed according to the following sequences: P_AtSMAP2-1993_ –F (CACCAAGCTGTTTTGAAATTTATATCCAG); P_AtSMAP2-997_ –F (CACCTTGACTTTGAAGTAAAAGTTTGGAC); and P_AtSMAP2-1993_/P_AtSMAP2-997_ –R (AACAAAACCTAAGGATAAAGAGAAAG). The PCR reactions were performed using *Arabidopsis* genomic DNA as a template, and the reaction volume was 50 µL. The PCR conditions for amplifying *AtSMAP2* promoters were as follows: 95 °C for 3 min; 35 cycles of 95 °C for 30 s, 60 °C for 30 s, 72 °C for 2 min; and then final extension at 72 °C for 5 min. After the PCR amplification product was recovered using a purification kit, ligated to the TOPO vector, and transformed into competent cells of *E. coli* TOP10 by the heat shock method, single colonies were picked at random, and the positive colonies were sent to the company for sequencing verification after colony PCR verification.

### Construction of expression vectors and genetic transformation of *Arabidopsis*

The correctly sequenced TOPO vector and the plant expression vector pMDC162 plasmid were digested separately and recombined using the ClonExpress^®^ II One Step Cloning Kit C112. The recombinant product was transformed into *E. coli* DB3.1 competent cells, and single colonies were randomly picked. After colony PCR verification, the positive colonies were sent to the company for sequencing verification.

The correct vector confirmed by sequencing is the constructed expression vector of the *AtSMAP2* promoter driving the *GUS* gene, named P_AtSMAP2-997_-GUS and P_AtSMAP2-1993_-GUS. The above recombinant vector was transformed into *Agrobacterium* strain GV3101 competent cells, and *Agrobacterium* colonies were identified by PCR to obtain GV3101 strains carrying P_AtSMAP2-997_-GUS and P_AtSMAP2-1993_-GUS. Flower buds of *Arabidopsis thaliana* Col-0 ecotype at the flowering stage were then infested with the above bacterial solution using the floral dipping method^36^ and the T1 generation seeds were obtained.

### Culture and identification of transgenic *Arabidopsis*

The seeds of *Arabidopsis* were first sterilized with 75% alcohol for 5 min, washed with sterile water, soaked in 30% NaClO for 10 min, and then washed with sterile water 5 to 6 times, and vernalized at 4 °C for 3 ~ 4 days. The T1 transformants were selected on the half-strength Murashige and Skoog (MS) plates containing hygromycin at a final concentration of 50 mg/L. And then these plants were cultured in a growth chamber with a light intensity of 120 μmol·m^−2^ s^−1^, a humidity of approximately 60%, 24 °C light for 16 h, and 20 °C darkness for 8 h. The regenerated T1 seedlings with hygromycin resistance were confirmed by PCR amplification using a *promoter-GUS* specific primer (P_AtSMAP2-1993_-GUS/P_AtSMAP2-997_-GUS-R: 5′CTGCTTTTTCTTGCCGTTTTCGTCG3′; P_AtSMAP2-1993_-GUS-F: 5′GTACTCTTGATCTTTATTACCTTGG3′, the length of the amplification product was 1524 bp; P_AtSMAP2-997_-GUS-F: 5′TGTTGATGACACATAAAGAAAGAAA3′, the length of the amplification product was 638 bp) and then transplanted into the soil and grown to maturity in a greenhouse. The T2-generation transgenic lines that displayed a segregation ratio of 3:1 by hygromycin resistance screening were used for propagation. Finally, the homozygous transgenic lines with a single insertion of the *AtSMAP2 promoter-GUS* were screened for functional analyses through segregation ratio analysis.

### Treatments with different concentrations of 2,4-D, IAA, ABA and MeJA

Wild-type (Col-0) and transgenic seedlings were grown on MS medium solidified with 0.8% agar in a growth chamber for 7 days. Twenty seedlings per transgenic line were used for each treatment. The hormone treatments were carried out for 12 h and involved culturing 7-day-old seedlings in liquid MS medium containing 50nM, 100nM, 150nM 2,4-D and IAA, 25µM, 50µM, 100µM ABA, 1µM, 25µM, and 50µM MeJA, respectively, while control seedlings were cultured in liquid MS medium without any hormones.

### GUS histochemical staining and quantitative real-time PCR (qRT‒PCR) analysis

According to the GUS staining kit, the transgenic *Arabidopsis* seedlings were completely immersed in GUS staining solution, incubated at 37 °C overnight, and then seedlings were moved to 75% ethanol for decolorization, observed for GUS staining, and photographed for preservation.

The total RNA of *Arabidopsis thaliana* was extracted with a Spectrum Plant Total RNA Kit (Sigma‒Aldrich, Germany), and reverse transcription synthesis was performed using StarScript II First-strand cDNA Synthesis Mix with gDNA Remover (GenStar, China). The enzyme used for qRT‒PCR was 2 × RealStar Green Fast Mixture with ROX (GenStar, China), and the equipment used was a StepOnePlus™ real-time PCR device (Thermo Fisher Scientific, Applied Biosystems, China). The primers for *GUS* were F: 5′- CGAACTGAACTGGCAGACTATCC-3′ and R: 5′- CGGCGTGGTGTAGAGCATTAC-3'; and the expression of *Actin2* was used as an internal control, the primers for *Actin2* were F: 5'-ACGAACTGAACTGGCAGACTATCC-3' and R: 5'-TCGGCGTGGTGTAGAGCATTAC-3'. Relative gene expression was calculated using the 2^−ΔΔCt^ method, with Ct values averaged over three technical replicates, and the relative expression was the average of three biological replicates.

## Data Availability

The datasets analysed during the current study are available in the The Arabidopsis Information Resource (TAIR) (BioSample number: AT3G24280), https://www.arabidopsis.org/servlets/TairObject?id=37851&type=locus.
